# Society Meetings

**Published:** 1918-11

**Authors:** 


### SOCIETY MEETINGS

Brief reports and announcements of meetings of societies of Western New York, and those of general scope, are requested from Secretaries. Copy should be on hand the fifteenth of the month. Full transactions will be published at cost of composition.

**The Medical Association of Central N. Y.** omitted its annual meeting in Oct. on account of the absence of large proportion of its members in military service. The Ohio State Society has taken similar action and it strikes us that it is sound economy both of money and effort to forego most medical meetings that require more than local travel, until the war is over.

**Rochester Academy of Medicine,** meeting; October 9th. Captain J. H. Elliott of Toronto read a paper on "Tuberculosis in the Canadian Forces and how the Problem is being Solved."

The annual meeting of the **New York and New England Association of Railway Surgeons** was held in New York on October 21st. Papers were read by Drs. W. H. Marcy of Buffalo; Donald Guthrie, Sayre, Pa.; W. L. Estes, South Bethlehem, Pa.; Walter E. Lee, Philadelphia; Chas. Geiger, St. Joseph; J. S. Hill, Bellows Falls, Vt.; Jos. C. Bloodgood, Baltimore; C. W. Hopkins, Chicago; Wm. Seiger, Pueblo, Col.; F. Park Lewis, Buffalo, and A. H. Harriman, Laconia, N. H.

The Annual Meeting of the **Medical Society of Central New York** scheduled to take place on 10-17-18 will not be held this year. This action was taken at a meeting of the executive committee who decided that because of absence of so many members at the front and account of the supreme importance of giving all available time to war matters, it was advisable to postpone the next annual meeting to October, 1919.

The first meeting of the season of the **Buffalo Academy of Medicine** was held October 2d. Dr. Wm. D. Johnson of Batavia read a paper on "Improvement in Surgical Results," and Dr. Bernard Schreiner read one on "Treatment of Basal-Celled Epithelioma, with report of 50 cases."

The second meeting was held on October 9th under the

Section of Medicine. Dr. Frederick Peterson of New York read a paper on "Preventive Medicine and the Reconstruction of the Race."

Section of Gynecology and Obstetrics—October 16. Program—"The Psycological Neurosis of Pregancy and the Peurperium," by Dr. Jennie Harris of North Tonawanda. "Treatment of Posterior Presentation," by Dr. Thomas Allen of Buffalo.

Section of Pathology—October 23. Program—"Value of Spinal Fluid Examination," by Dr. John Eckel of Buffalo.

Section of Surgery—November 6th. "The Skin Sliding Operations in Chronic Suppurative Diseases, especially in Empyema," by Dr. Emil G. Beck of Chicago.

All members entering military service may have their dues refunded. Those in service are exempt from payment of dues and will be considered in good standing until their return to civil practice.

